# Spatiotemporal dynamics of *Bacillus anthracis* under climate change: a machine learning approach

**DOI:** 10.3389/fmicb.2025.1659876

**Published:** 2025-10-14

**Authors:** Sameh M. H. Khalaf, Monerah S. M. Alqahtani, Yousef A. Selim, Kenoz O. Elsayed, Hager A. Bendary

**Affiliations:** ^1^Faculty of Biotechnology, October University for Modern Sciences and Arts (MSA University), 6th October City, Egypt; ^2^Department of Biology, Faculty of Science, King Khalid University, Abha, Saudi Arabia; ^3^Department of Microbiology and Immunology, Faculty of Pharmacy (Girls), Al-Azhar University, Cairo, Egypt

**Keywords:** *Bacillus anthracis*, species distribution modeling, climate change, ecological niche, epidemiology

## Abstract

This study examines the spatiotemporal dynamics of *Bacillus anthracis*, the causative agent of anthrax, under climate change scenarios using advanced machine learning techniques. Climate change is increasingly recognized as a critical factor influencing the distribution and transmission dynamics of infectious diseases, particularly those reliant on environmental reservoirs. Our research employs Maximum Entropy (Maxent) modeling to forecast the current global distribution of *B. anthracis* based on climatic factors and to predict future habitat suitability under various Coupled Model Intercomparison Project Phase 5 (CMIP5) scenarios (RCP-2.6 and RCP-8.5) for the 2050’s and 2070’s. We identify high-risk areas where climate change may enhance the suitability for *B. anthracis*, emphasizing the need for proactive monitoring and early-warning systems. The findings indicate potential shifts in anthrax-endemic zones, with new regions becoming conducive to the establishment of *B. anthracis* due to the changing climate. Our results demonstrate the applicability of machine learning in predicting disease risk, providing a framework for public health preparedness in light of evolving environmental challenges. These insights are critical for developing targeted surveillance strategies and mitigating the introduction of zoonotic diseases in a warming environment.

## Introduction

Climate change is widely acknowledged as a significant factor influencing the distribution and transmission dynamics of infectious illnesses, especially those reliant on environmental reservoirs or vector-dependent routes (Rocklöv and Dubrow, 2020; Franklinos et al., 2019). Anthrax, caused by the spore-forming bacterium *Bacillus anthracis*, constitutes a considerable zoonotic risk with intricate ecological interdependencies. The spore is the infectious form and its ability to survive in soil, along with its need on particular climatic and soil conditions, renders its epidemiology acutely responsive to environmental alterations (Hugh-Jones and Blackburn, 2009; Carlson et al., 2019). Emerging anthrax cases in regions previously considered non-endemic have raised concerns about climate-driven range expansion of *B. anthracis*, necessitating advanced predictive models to anticipate future distribution shifts (Mullins et al., 2021; Waits et al., 2018).

The ecological niche of *B. anthracis* is influenced by various biotic and abiotic variables, such as soil pH, organic carbon content, temperature, and precipitation patterns (Dragon and Rennie, 1995; Blackburn et al., 2017). These variables affect spore viability, host exposure, and outbreak prevalence, resulting in specific spatial hotspots where environmental conditions promote disease persistence (Fasanella et al., 2013; Kracalik et al., 2017). As global temperatures increase and precipitation patterns change due to climate change, classic anthrax-endemic zones may undergo modified transmission dynamics, while new areas may become conducive to the establishment of *B. anthracis* (Elith and Leathwick, 2009; Escobar and Craft, 2016). Comprehending these transitions is essential for proactive monitoring and reducing spillover into human and animal populations.

Species distribution models (SDMs) are essential instruments in epidemiology for forecasting habitat appropriateness amid evolving environmental variables (Peterson et al., 2011; Franklin, 2013). Maximum Entropy (Maxent) modeling has acquired recognition for its efficacy in managing presence-only occurrence data and incorporating intricate environmental factors (Phillips et al., 2006; Merow et al., 2013). Maxent’s machine learning framework facilitates accurate predictions despite sparse occurrence records, rendering it especially useful for modeling diseases such as anthrax, which frequently exhibit uneven and underreported dispersion data (Elith et al., 2011; Sofaer et al., 2019). Recent utilizations of Maxent in disease ecology have effectively forecasted range shifts for vector-borne and soil-borne diseases, underscoring its applicability in climate change impact assessments (Escobar et al., 2016; Carlson et al., 2022).

Notwithstanding these advancements, limited research has utilized machine learning techniques to predict the future distribution of *B. anthracis* in the context of climate change scenarios. Prior endeavors predominantly depended on static ecological niche models or concentrated on regional-scale dynamics (Blackburn et al., 2007; Mullins et al., 2015). The rapid progression of climate change necessitates high-resolution, global-scale forecasts to guide public health preparedness (IPCC, 2021; Mora et al., 2022). The Coupled Model Intercomparison Project Phase 5 (CMIP5) offers revised climate forecasts based on Representative Concentration Pathways (RCPs), enabling the evaluation of *B. anthracis* distribution across several future scenarios (Eyring et al., 2016; Warszawski et al., 2021). Combining these data with machine learning methodologies can produce more precise and actionable forecasts for disease risk mapping.

The interaction between climate change and land-use alterations may further influence anthrax transmission by modifying wildlife-livestock-human interactions (Hansen et al., 2019; Walsh et al., 2022). Deforestation, agricultural development, and urbanization may disturb soil ecosystems, thereby heightening human exposure to *B. anthracis* spores in emerging hotspots (Barro et al., 2016; Bezymennyi et al., 2021). A spatially explicit modeling technique that incorporates both climatic and anthropogenic factors is needed for thorough risk assessment. This study seeks to address significant gaps in comprehending the spatiotemporal dynamics of anthrax under future warming conditions by utilizing high-resolution environmental datasets and sophisticated machine learning methods.

This study utilizes Maxent modeling to (1) estimate the present global distribution of *B. anthracis* based on climatic factors; (2) anticipate future habitat suitability under CMIP5 climate scenarios (RCP-2.6 and RCP-8.5) for the 2050’s and 2070’s; and (3) pinpoint high-risk areas where climate change may enhance anthrax suitability. Our findings will establish a basis for focused surveillance and early-warning systems in at-risk ecosystems, thereby enhancing One Health measures to mitigate the introduction of zoonotic diseases in a warming environment.

## Materials and methods

### Occurrence data and preprocessing

**FIGURE 1:**
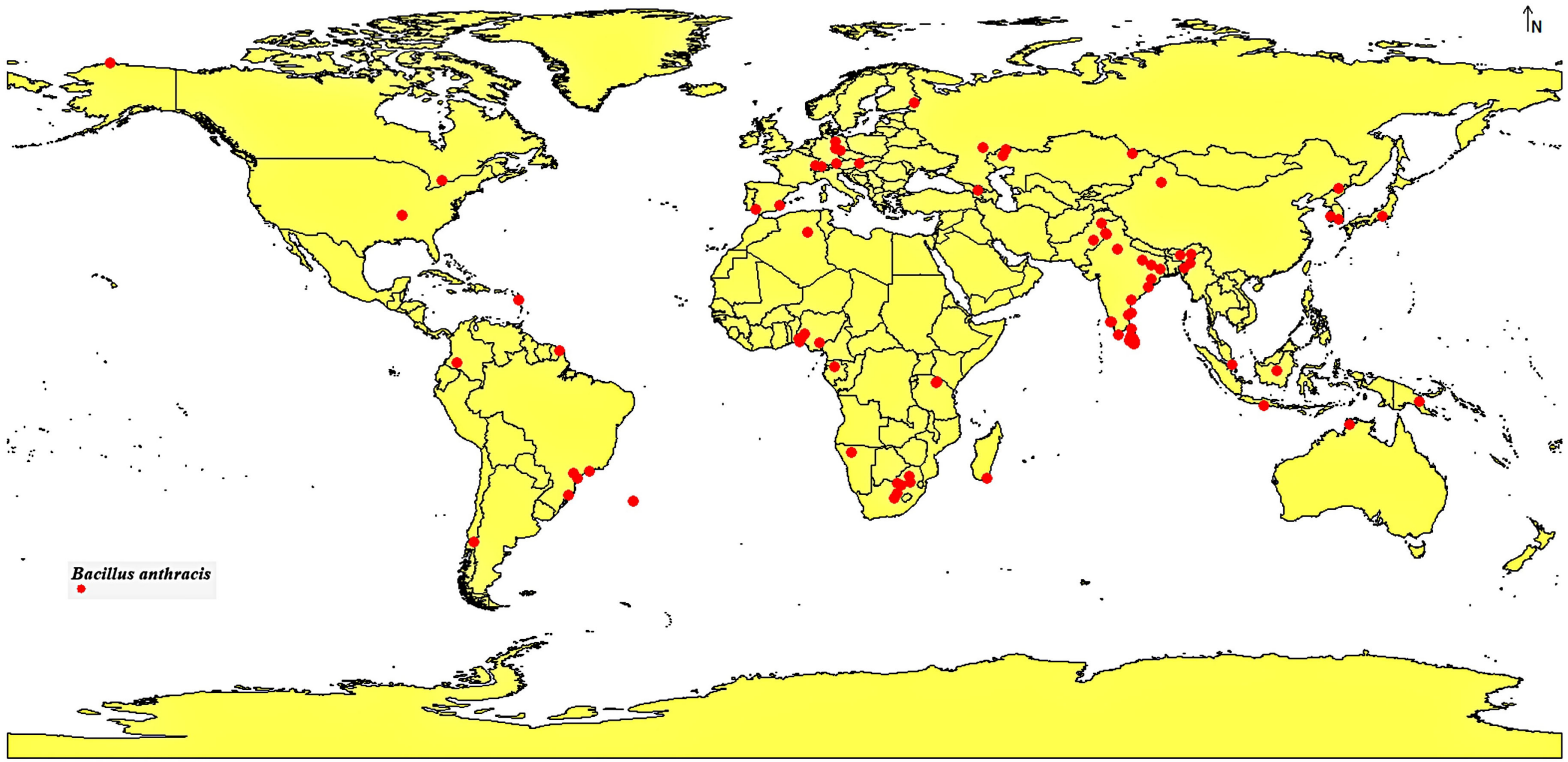
Sites of *B. anthracis* occurrence used in this study.

The occurrence data for *B. anthracis* were aggregated from the Global Biodiversity Information Facility (GBIF) and documented anthrax outbreak records (Anonymous, 2025), resulting in an initial dataset of 733 georeferenced sites. To guarantee data integrity, duplicates were eliminated, and spatially ambiguous records (more than 1 km uncertainty) were discarded. Spatial rarefaction was implemented using ArcGIS Pro v3.1 to reduce sampling bias and prevent clamping, yielding 105 high-confidence occurrence sites. The filtered records were exported in CSV format for future modeling (Figure 1 and Supplementary file 1).

### Environmental factors and selection

Nineteen bioclimatic variables with a geographical resolution of approximately 5 km^2^ were sourced from WorldClim (version 2.1), reflecting long-term climatic averages from 1970 to 2000 (Hijmans, 2017). To mitigate multicollinearity, a Pearson correlation analysis (|r| > 0.8) was performed in R v4.3.0, preserving five biologically pertinent predictors including evaluation of the 19 bioclimatic variables using pre model to excluded the factors that have no or a minimal effect on distribution of this species: BIO 2 (Mean Diurnal Range), BIO 6 (Minimum Temperature of Coldest Month), BIO 7 (Temperature Annual Range), BIO 16 (Precipitation of Wettest Quarter), and BIO 17 (Precipitation of Driest Quarter) were finally selected. Future climate forecasts for 2050 and 2070 were obtained from CMIP5 under two Representative Concentration Pathway RCPs 2.6 & 8.5 and downscaled to align with WorldClim’s resolution (Alkhalifah et al., 2022).

Ecological Niche Modeling Habitat appropriateness for *B. anthracis* was assessed utilizing Maxent v3.4.4, a presence-background machine learning approach (Phillips et al., 2006). The model was trained on 75% of the occurrence data, while the remaining 25% was allocated for testing. Essential parameters comprised 10,000 background points, 1,000 iterations, and 10-fold cross-validation to augment robustness (Merow et al., 2013). Linear, quadratic, and hinge feature classes were utilized to identify potential non-linear interactions between the species and environmental variables. A BIOCLIM model was constructed in DIVA-GIS v7.5 for comparative analysis (Hijmans et al., 2012), utilizing the same occurrence data and factors to produce an environmental envelope-based prediction.

### Model validation and performance metrics

The model’s performance was assessed using the Area Under the Curve (AUC) metric, with values exceeding 0.8 signifying robust predictive accuracy (Booth et al., 2014; Hosni et al., 2020). The influence of each bioclimatic variable was evaluated using a jackknife test, and response curves were produced to illustrate the species-environment interactions. The True Skill Statistic (TSS) was computed to further assess model performance, with values exceeding 0.6 being acceptable (Alkhalifah et al., 2023).

### Visualization and thresholding

Habitat suitability maps were generated in ArcGIS Pro, with suitability scores categorized using Jenks natural breaks into five classifications: Unsuitable (< 0.2), Low (0.2–0.4), Moderate (0.4–0.6), High (0.6–0.8), and Excellent (> 0.8). Future predictions were transformed into binary presence/absence maps with a threshold derived from the maximal training sensitivity combined with specificity (Alqahtani et al., 2025). Maps illustrating changes were produced to represent alterations in appropriateness under prospective climatic scenarios, classified as gain, loss, or unchanged (Khalaf et al., 2024).

### Analysis of limiting factors and ecological niches

A two-dimensional niche envelope test was conducted in DIVA-GIS to ascertain the principal restrictions on *B. anthracis* distribution, concentrating on BIO 1 (Annual Mean Temperature) and BIO 12 (Annual Precipitation). This investigation defined the environmental limits within which the species is expected to survive. A limiting factor map was generated to identify areas where particular bioclimatic factors (e.g., BIO 6 or BIO 17) had the most significant impact on habitat suitability (Peterson et al., 2011).

## Results

### Bioclimatic factor correlation analysis and bioclimatic factor selection

**FIGURE 2:**
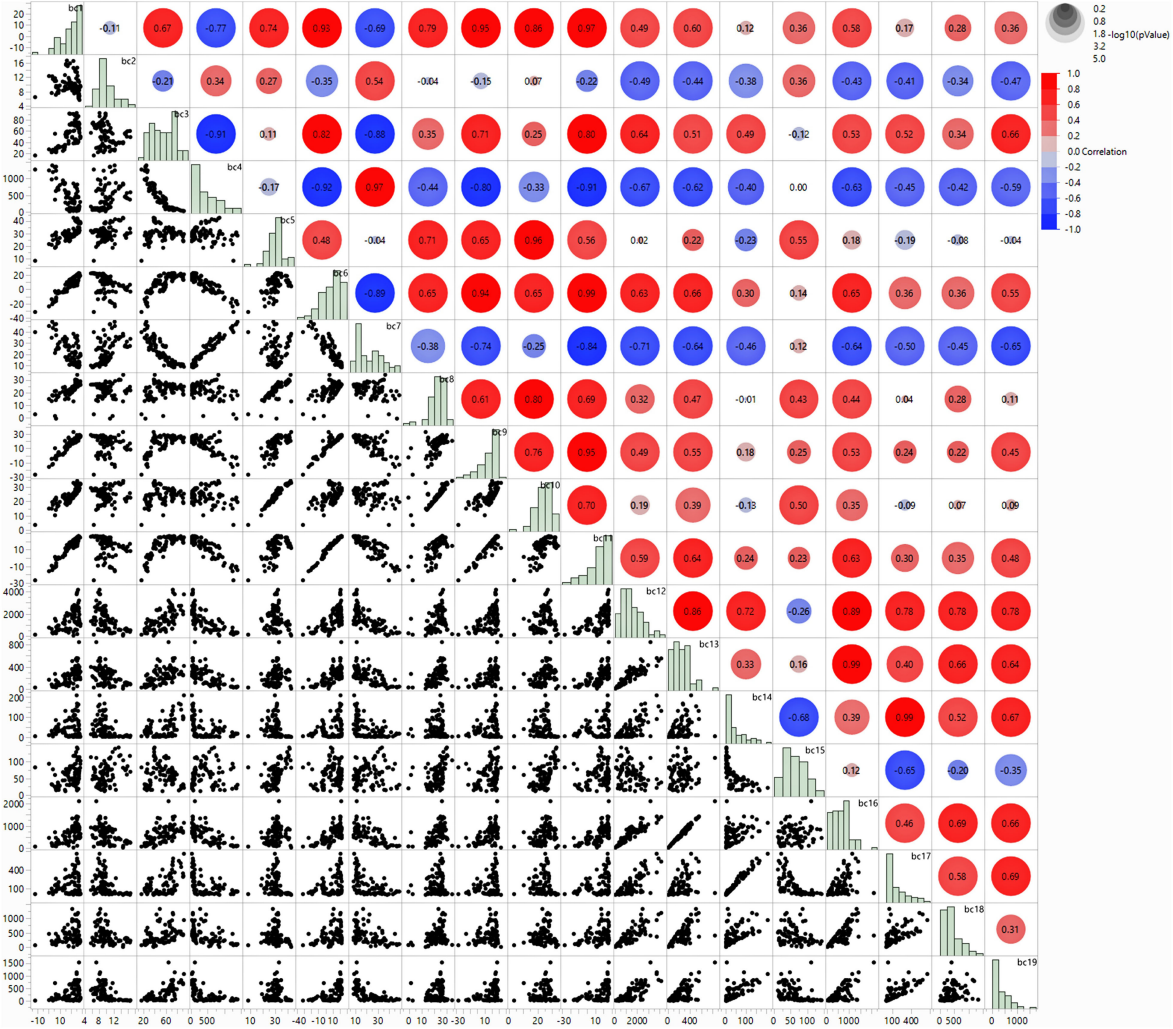
Correlation matrix and scatter plot analysis of 19 bioclimatic variables used in species distribution modeling for *B. anthracis*. The upper triangle shows Pearson correlation coefficients with color coding (red, positive correlation; blue, negative correlation), while the lower triangle displays scatter plots illustrating pairwise relationships between variables. Diagonal elements show frequency distributions for each bioclimatic variable. Circle size and color intensity indicate correlation strength, with larger, more intensely colored circles representing stronger correlations (|r| > 0.8 threshold used for variable selection).

To address multicollinearity concerns that may undermine model efficacy and interpretation, we performed an extensive Pearson correlation study of all 19 bioclimatic variables, employing the criterion of |r| > 0.8 as advised for species distribution modeling (Dormann et al., 2013). The correlation matrix indicated significant multicollinearity among many bioclimatic variables, requiring meticulous variable selection to preserve only ecologically relevant and statistically independent predictors (Figure 2).

The correlation analysis revealed several clusters of highly associated variables. BIO_1 (Annual Mean Temperature) had robust positive correlations with BIO_6 (*r* = 0.93), BIO_9 (*r* = 0.95), BIO_10 (*r* = 0.86), and BIO_11 (*r* = 0.97), suggesting that these temperature-related variables mostly conveyed redundant information. Likewise, precipitation variables demonstrated significant intercorrelations, with BIO_13 (Precipitation of Wettest Month) exhibiting a strong correlation with BIO16 (*r* = 0.99), while BIO12 (Annual Precipitation) displayed considerable correlations with several precipitation variables (BIO_16: *r* = 0.89, BIO17: *r* = 0.78, BIO18: *r* = 0.78, BIO19: *r* = 0.78).

Temperature seasonality variables exhibited significant multicollinearity, as BIO_3 (Isothermality) and BIO4 (Temperature Seasonality) displayed a strong negative correlation (*r* = −0.91), while both variables also showed high correlations with BIO_7 (Temperature Annual Range): BIO3 (*r* = 0.82) and BIO_4 (*r* = −0.81). Furthermore, the extreme temperature variables BIO5 (Maximum Temperature of the Warmest Month) and BIO_6 (Minimum Temperature of the Coldest Month) exhibited a strong correlation with annual temperature metrics.

Subsequent to the multicollinearity evaluation, we employed ecological relevance criteria pertinent to *B. anthracis* spore biology to identify five bioclimatic factors that were both statistically independent (|r| < 0.8) and biologically significant:

BIO_2 (Mean Diurnal Range) was retained because to its relatively low correlations with other selected variables and its representation of daily temperature swings essential for spore survival and germination processes. Diurnal temperature fluctuations influence spore metabolic activity and stress resilience, rendering this variable crucial for forecasting viable spore longevity.

BIO_6 (Minimum Temperature of Coldest Month) was chosen despite its association with temperature factors, as extreme cold tolerance is a critical limiting factor for *B. anthracis* spore survival. This variable denotes the essential lower temperature threshold beneath which spore survival rates markedly decrease, crucial for forecasting distribution limitations at elevated latitudes and altitudes.

BIO_7 (Temperature Annual Range) denotes seasonal temperature variations, affecting spore dormancy cycles and germination timing. This variable demonstrated adequate correlation levels with our other chosen predictors while encapsulating the annual temperature amplitude that influences long-term spore persistence in soil conditions.

BIO_16 (Precipitation of Wettest Quarter) was selected to signify moisture availability during peak precipitation periods, influencing soil water content essential for spore germination in the animal host, and host exposure risk. Although associated with some precipitation variables, it retained statistical independence from our chosen temperature predictors and reflects seasonal moisture peaks pertinent to *B. anthracis* ecology.

BIO_17 (Precipitation of Driest Quarter) indicates water stress circumstances during arid intervals, essential for comprehending spore viability under moisture constraints. This measure enhances BIO16 by quantifying moisture availability and exhibited the weakest relationships with our chosen temperature variables (BIO_2: *r* = −0.41, BIO_6: *r* = 0.36, BIO_7: *r* = −0.50).

The conclusive correlation study of the five chosen variables validated effective multicollinearity reduction, with all pairwise correlations remaining beneath the |r| = 0.8 threshold. The most significant association among the chosen variables was between BIO_6 and BIO_7 (*r* = −0.69), followed by BIO_16 and BIO_17 (*r* = 0.46), demonstrating adequate statistical independence while preserving biological significance for *B. anthracis* ecological niche modeling.

### Model validation and climatological factors influence

**FIGURE 3:**
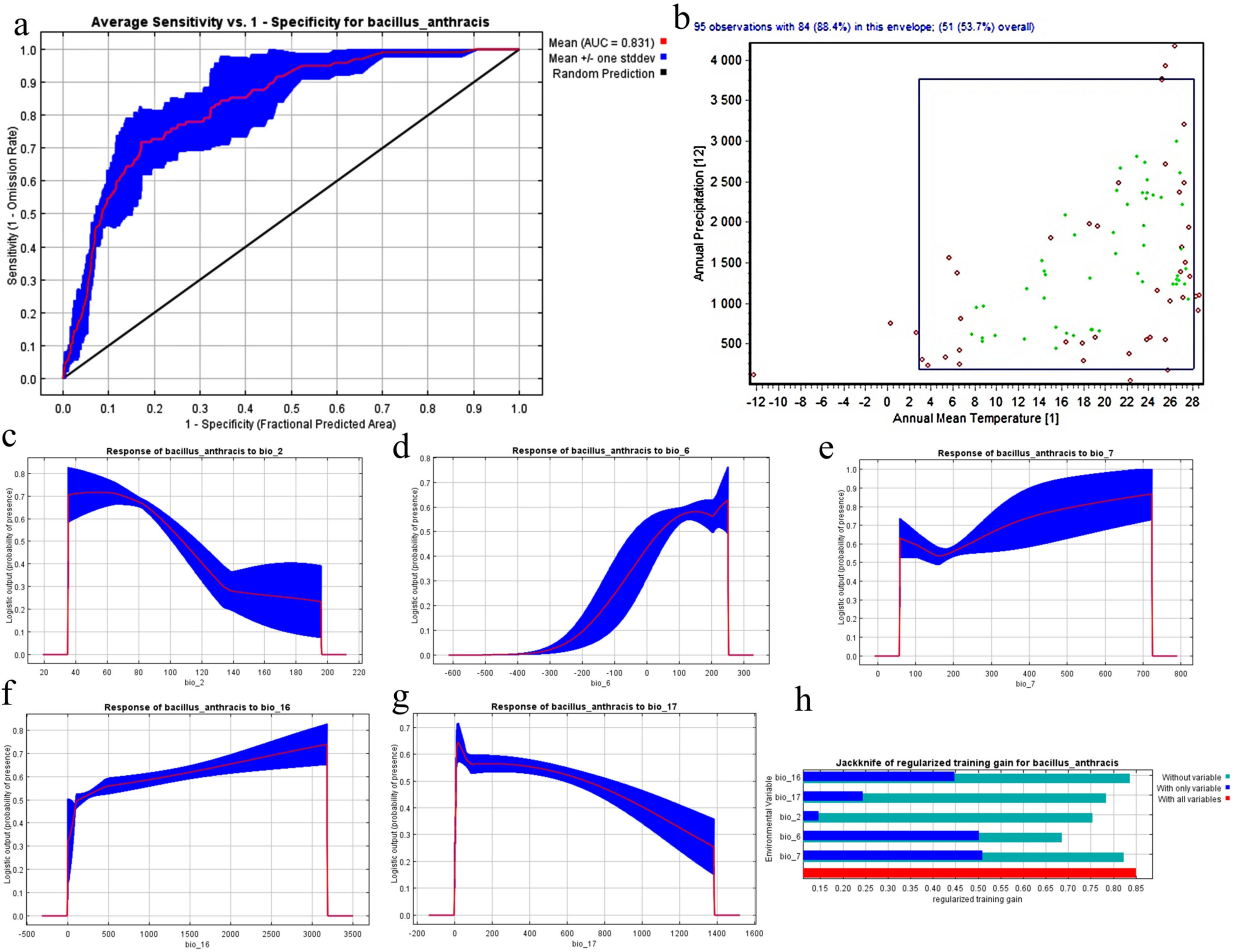
Maxent model validation and response curves for *B. anthracis* **(a)** Receiver Operating Characteristic (ROC) curve showing the average test sensitivity versus 1-specificity for the Maxent model, with an Area Under the Curve (AUC) of 0.831 indicating strong predictive performance. **(b)** Two-dimensional niche analysis illustrating the environmental envelope of *B. anthracis* occurrences, based on Annual Mean Temperature (BIO_1) and Annual Precipitation (BIO_12). **(c–g)** Response curves depicting the relationship between logistic suitability for *B. anthracis* and five key bioclimatic variables: **(c)** Mean Diurnal Range (BIO_2), **(d)** Minimum Temperature of Coldest Month (BIO_6), **(e)** Temperature Annual Range (BIO_7), **(f)** Precipitation of Wettest Quarter (BIO_16), and **(g)** Precipitation of Driest Quarter (BIO_17). The red lines represent the mean response, and the blue shaded areas indicate +/– one standard deviation. **(h)** Jackknife test of variable importance, showing the gain achieved by using each variable alone, by excluding each variable, and by using all variables, highlighting their contribution to the model’s predictive power.

The predictive power and ecological insights gleaned from our Maxent model performance for *B. anthracis* was meticulously validated using the Receiver Operating Characteristic (ROC) curve, illustrated in Figure 3a, resulting in an Area Under the Curve (AUC) value of 0.831. The substantial AUC value, markedly above random prediction (AUC = 0.5), demonstrates the model’s excellent capacity to differentiate between the presence and absence of *B. anthracis* sites, hence instilling high confidence in its predictive accuracy. The TSS value was 0.78, which indicates the good accuracy of the generated model.

A comprehensive understanding of the environmental niche of *B. anthracis* was attained using a two-dimensional niche analysis, illustrated in Figure 3b, which examines the correlation between Annual Mean Temperature (BIO_1) and Annual Precipitation (BIO_12). This scatter figure depicts the environmental conditions in which *B. anthracis* occurrences are predominantly located inside a designated envelope (The red color indicated occurrence of this records outside the enveloped either for this two variables or when test any other variables (red points inside the current enveloped), while green points indicate occurrence of these records inside enveloped either for these two variables or any other variables of 19 bioclim), implying certain ideal ranges for these two essential climatic variables. The bulk of recorded instances are concentrated within a limited range of annual mean temperatures (about 5 °C–28 °C) and yearly precipitation (from around 500 to 3,000 mm), while some instances exceed these parameters, underscoring the bacterium’s flexibility.

The response curves for the five most significant bioclimatic variables affecting *B. anthracis* adaptability are illustrated in Figures 3c–g. Figure 3c for BIO_2 (Mean Diurnal Range) illustrates a complex correlation, indicating that appropriateness often diminishes as the diurnal temperature range increases, implying that *B. anthracis* favors habitats with more stable daily temperatures. The response to BIO 6 (Minimum Temperature of Coldest Month), illustrated in Figure 3d, demonstrates enhanced suitability with elevated minimum temperatures during the coldest month, affirming that extreme cold is a critical limiting factor. Figure 3e, depicting the response to BIO_7 (Temperature Annual Range), indicates that *B. anthracis* spore adaptability is maximized at moderate levels of annual temperature variability, decreasing in habitats characterized by either little or excessive annual temperature swings. Figure 3f (BIO_16: Precipitation of Wettest Quarter) illustrates that suitability first rises with precipitation during the wettest quarter, but then either levels off or slightly declines at elevated levels, possibly indicating a threshold for ideal moisture. In contrast, Figure 3g (BIO_17: Precipitation of Driest Quarter) illustrates that suitability is typically greater in regions with some moisture during the driest quarter, while extremely low precipitation levels are detrimental, underscoring the significance of year-round moisture availability to a certain extent.

The Jackknife test (Figure 3h) elucidated the relative significance of each environmental variable to the model’s efficacy. This investigation indicates that the variables BIO_17 (Precipitation of Driest Quarter), BIO_2 (Mean Diurnal Range), and BIO_6 (Minimum Temperature of Coldest Month) each significantly enhance the model’s performance when utilized independently, demonstrating their robust predictive capability. Moreover, the exclusion of each variable reveals a decline in regularization training gain, underscoring their distinct informational value and affirming that these variables contribute essential and non-redundant insights for the precise modeling of *B. anthracis* distribution. These results collectively validate the robustness of our Maxent model and reveal the principal environmental factors influencing the worldwide ecological niche of *B. anthracis*.

### Current situation modeling

**FIGURE 4:**
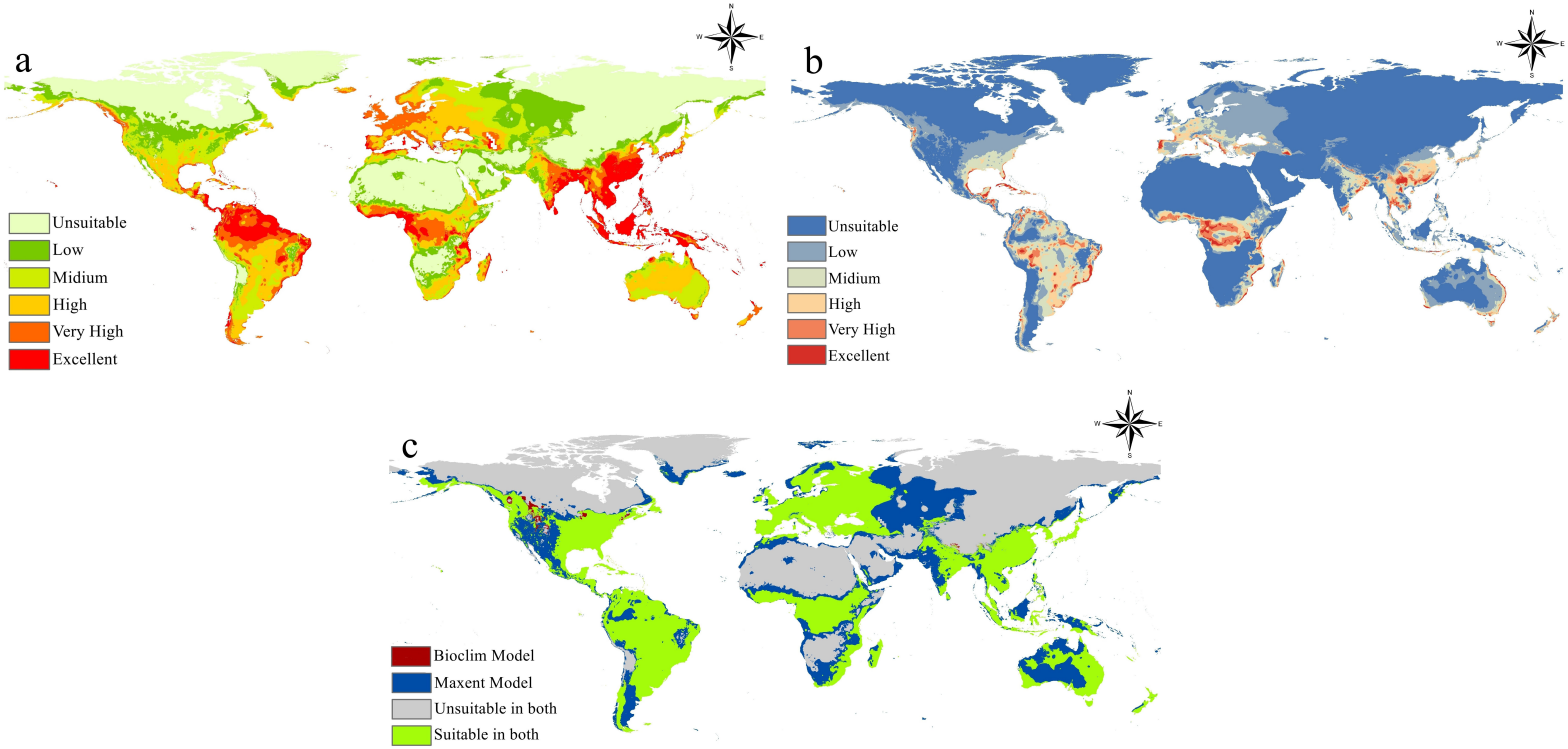
**(a)** Predicted global suitability for *B. anthracis* using the Maxent ecological niche model. **(b)** Predicted global suitability for *B. anthracis* using the BIOCLIM model from DIVA-GIS. **(c)** Comparison map showing areas of agreement and disagreement between the Maxent and BIOCLIM models.

The current spread of *B. anthracis* was evaluated with two separate species distribution modeling techniques: Maxent and BIOCLIM, with the resultant suitability maps and their discrepancies illustrated in Figure 4. The Maxent model (Figure 4a) defined a global distribution of *B. anthracis* suitability, indicating “Excellent” and “Very High” suitability in particular biological zones. The zones notably encompass the center and southern United States, especially the Great Plains, vast expanses of South America (e.g., Brazil, Argentina), substantial swaths of sub-Saharan Africa, and considerable parts of center Asia, India, and Southeast Asia. In contrast, regions at elevated latitudes, extensive deserts, and high-altitude landscapes repeatedly shown unfavorable or inadequate circumstances for the bacterium. This pattern corresponds with established environmental parameters affecting *B. anthracis* spore longevity, including alkaline, calcium-rich soils and particular climatic conditions typically seen in savanna-like or temperate grassland ecosystems.

Conversely, the BIOCLIM model (Figure 4b), executed via DIVA-GIS, typically forecasted a more limited range of appropriate habitat, identifying extensive areas as “Unsuitable” in northern latitudes and certain tropical forest regions such as the Amazon. Although BIOCLIM detected “Excellent” and “Very High” suitability zones, these zones seemed more fragmented compared to those produced by Maxent, suggesting a more restricted definition of the environmental envelope favorable to *B. anthracis*. Notwithstanding these discrepancies, significant regions of high suitability consistently appeared in both models, including areas such as the center and southern United States, portions of South America, and different sites across Africa and Asia. The prevalent areas indicate a robust consensus on the essential environmental conditions that facilitate the persistence of *B. anthracis* spores in these places.

The direct comparison of the two models (Figure 4c) clearly delineates regions of agreement and disagreement. The most notable discovery is the extensive “Suitable in Both” category (green zones), which indicates habitats where both Maxent and BIOCLIM agree on the appropriateness for spores of *B. anthracis*. These regions signify the most reliable forecasts for the bacterium’s ecological niche, closely aligning with established anthrax endemic areas worldwide, including the central United States, some South American plains, extensive sub-Saharan Africa, and portions of Central and South Asia. The existence of “Maxent Model Only” (red) and “BIOCLIM Model Only” (blue) regions, however, limited in scope, highlights the fundamental algorithmic disparities between the two models. Maxent, a presence-background machine learning algorithm, is recognized for its capacity to elucidate intricate non-linear correlations between species occurrences and environmental variables, perhaps revealing appropriate locations overlooked by the more simplistic, climate-envelope-based BIOCLIM model. In contrast, BIOCLIM, by establishing suitability according to the spectrum of environmental variables at occurrence locations, may occasionally encompass wider appropriate areas or exhibit more sensitivity to anomalies in occurrence data. The significant overlap in forecasted appropriate areas among these several modeling methodologies enhances confidence in pinpointing high-risk zones for *B. anthracis* spores persistence and related anthrax outbreaks.

### Future prediction for *B. anthracis* under different climate change scenarios

**FIGURE 5:**
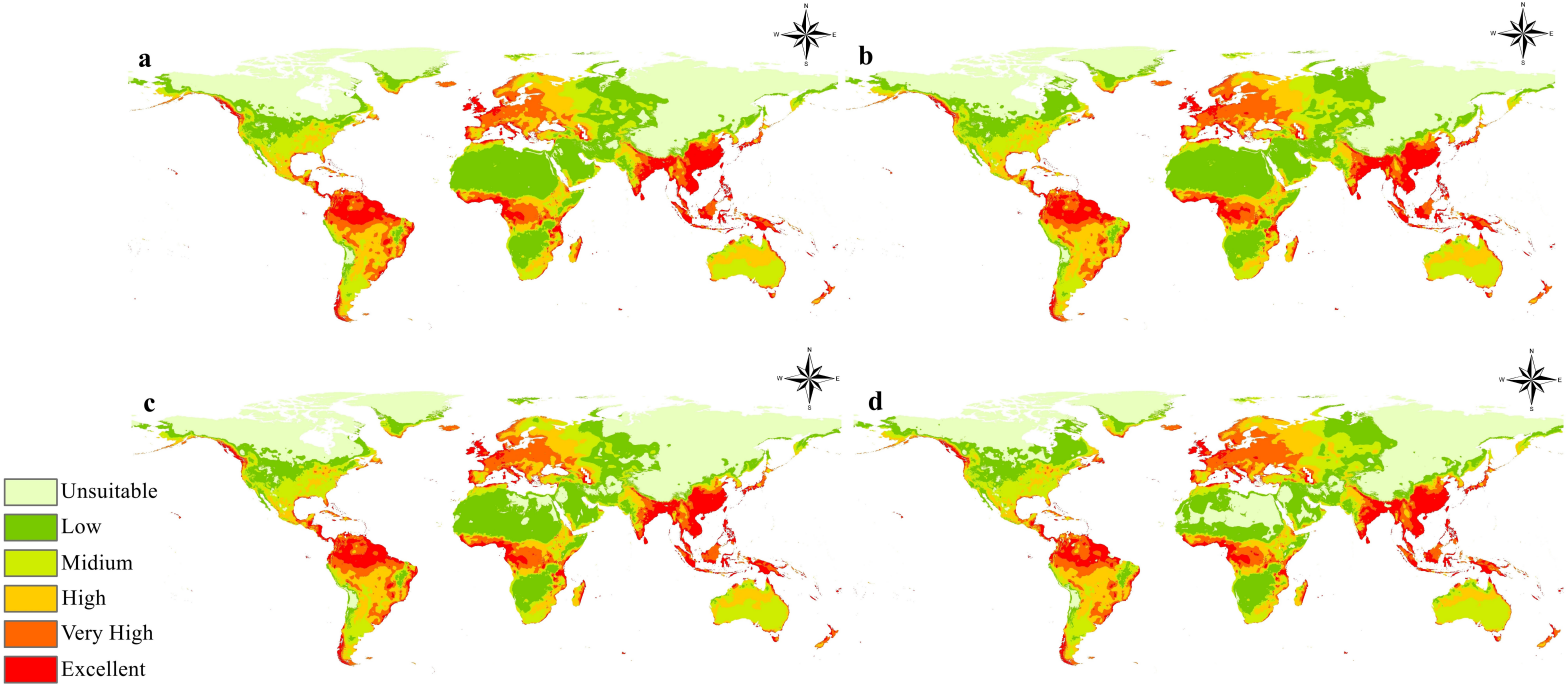
Global suitability maps for *B. anthracis* projected for different future climate scenarios: **(a)** 2050 under Representative Concentration Pathway (RCP) 2.6, **(b)** 2050 under RCP 8.5, **(c)** 2070 under RCP 2.6, and **(d)** 2070 under RCP 8.5.

The results of the MaxEnt modeling of four different climate change scenarios indicate changes in the overall habitat suitability of this bacterium. These forecasts are derived from several Representative Concentration Pathways (RCPs) and temporal frameworks, providing insights into the fluctuating characteristics of *B. anthracis’*s probable ecological niche. Figure 5 illustrates the anticipated global distribution of *B. anthracis* adaptability across four future climate change scenarios: (a) 2050 RCP 2.6, (b) 2050 RCP 8.5, (c) 2070 RCP 2.6, and (d) 2070 RCP 8.5. In all scenarios, a uniform pattern of high appropriateness, denoted by “Very High” and “Excellent” in orange and red, respectively, is evident in regions that predominantly correspond with the currently known suitable locations depicted in Figure 3. This encompasses substantial regions of the central and southern United States, South America (notably Brazil and Argentina), vast territories throughout sub-Saharan Africa, as well as sections of Central Asia, India, and Southeast Asia.

However, distinctions become apparent when contrasting the instances. In the more optimistic RCP 2.6 scenarios (Figures 5a, c), which presuppose a comparatively lower trajectory of greenhouse gas emissions, the spatial extent of highly appropriate places is largely analogous to the current distribution, with certain localized expansions or contractions. In contrast, the more gloomy RCP 8.5 scenarios (Figures 5b, d), indicative of elevated emission pathways, suggest a marginal shift or intensification of high suitability in certain places, alongside probable declines in others. Certain regions in Europe and Asia exhibit differing levels of appropriateness alterations across circumstances. The ongoing existence of extensive “Unsuitable” (light green) regions in northern latitudes, deserts, and high-altitude zones across all future scenarios indicates that these essential environmental limitations on *B. anthracis* distribution are expected to endure despite climate change.

**FIGURE 6:**
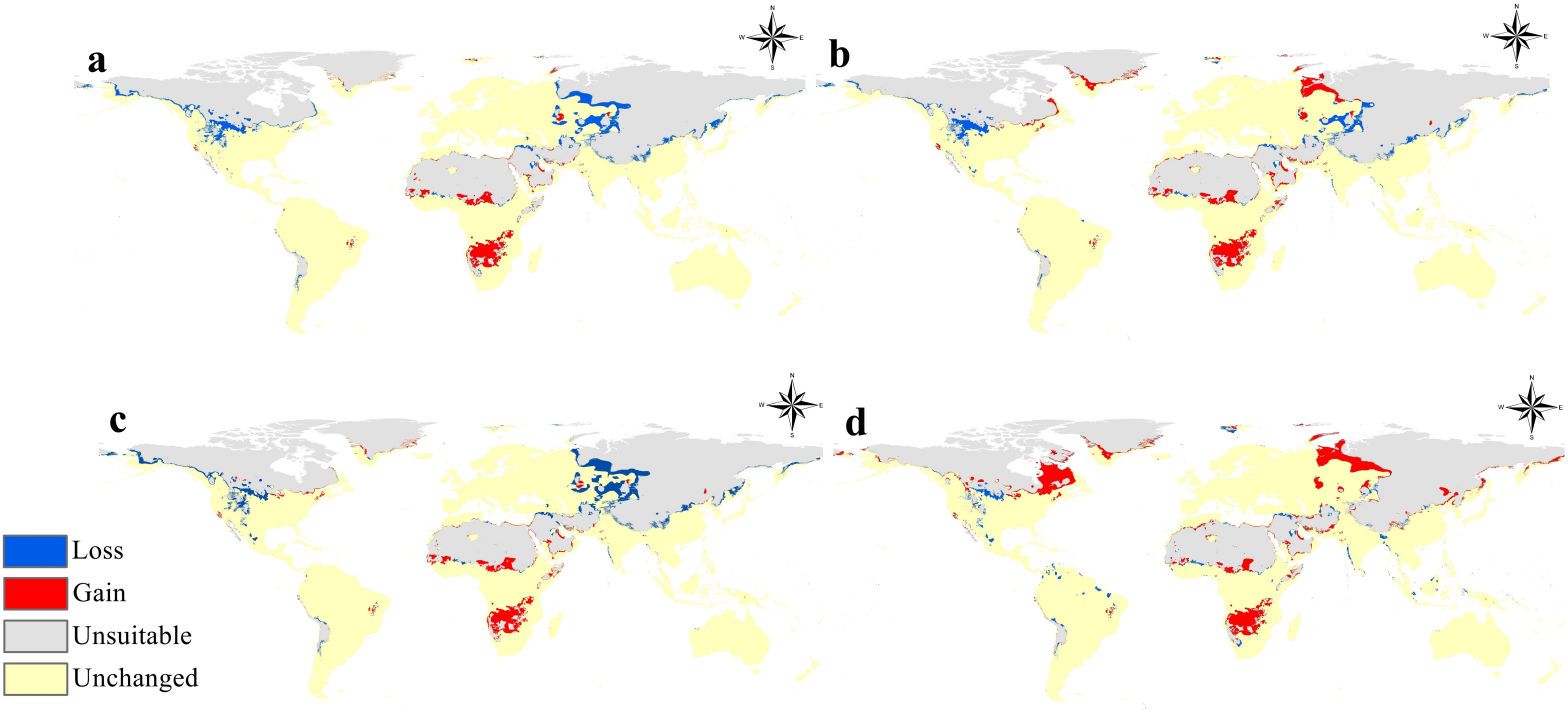
Global maps illustrating the projected changes in *B. anthracis* distribution compared to the current situation under future climate change scenarios: **(a)** 2050 under Representative Concentration Pathway (RCP) 2.6, **(b)** 2050 under RCP 8.5, **(c)** 2070 under RCP 2.6, and **(d)** 2070 under RCP 8.5.

On the other hand, Figure 6 clearly depicts the anticipated alterations in the acceptable habitat for *B. anthracis* under four distinct climate change scenarios: (a) 2050 RCP 2.6, (b) 2050 RCP 8.5, (c) 2070 RCP 2.6, and (d) 2070 RCP 8.5. This diagram classifies regions as “Loss” (blue), “Gain” (red), “Unsuitable” (gray), and “Unchanged” (yellow). A notable observation in all scenarios is the extensive prevalence of “Unchanged” (yellow) areas, especially in the central and southern sections of continents where *B. anthracis* is presently prevalent. This indicates that a significant fraction of the already suitable habitat is anticipated to remain constant under forthcoming climate circumstances, highlighting the durability of the pathogen’s niche in these established endemic regions.

Nonetheless, substantial “Gain” (red) in appropriate habitat is consistently anticipated in several critical regions across all scenarios, particularly in portions of North America (e.g., the western United States), southern South America, sections of southern and eastern Africa, and dispersed areas in Central Asia and Australia. These increases suggest a possible expansion of the *B. anthracis* ecological niche into places that were previously less appropriate or unsuitable. The magnitude and distribution of these improvements exhibit variability among RCPs and temporal frameworks, with the RCP 8.5 scenarios (Figures 5b, d) frequently demonstrating more significant gains in comparison to the RCP 2.6 scenarios. This indicates that elevated emission trajectories may facilitate increased opportunities for niche development. Conversely, the “Loss” (blue) of appropriate habitat is anticipated, but often in smaller and more isolated areas relative to gains. These losses are predominantly noted in places including portions of northern North America, some areas in Europe, and several sites in Asia. The occurrence of losses suggests that certain currently acceptable regions may become less viable under future climatic conditions, maybe due to alterations in precipitation patterns or temperature extremes surpassing the pathogen’s tolerance thresholds. Areas deemed “unsuitable” (gray), indicating regions unfit under both present and future conditions, predominantly align with extreme northern and southern latitudes, in addition to significant desert and mountainous terrains.

### Limitation factors map

**FIGURE 7:**
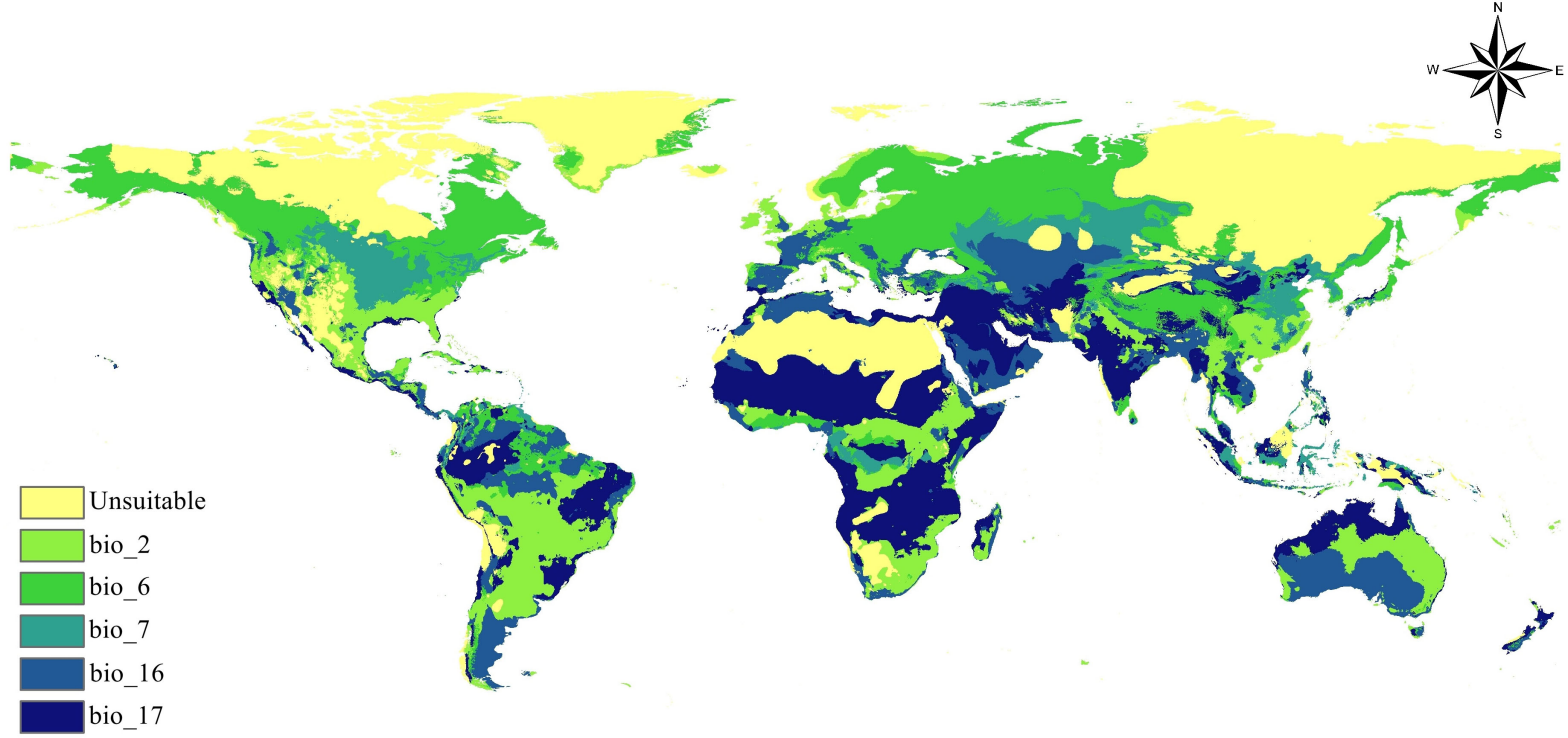
Limiting bioclimatic factors governing *B. anthracis* distribution.

Figure 7 depicts the primary bioclimatic conditions that substantially restrict the global distribution of *B. anthracis*. This map highlights the key environmental variables that, when deviating from ideal ranges, limit the bacterium’s distribution within its prospective geographic range. The map classifies locations according to the bioclimatic variable that has the most significant limiting effect. The regional variability of limiting bioclimatic parameters highlights the intricate interaction of environmental variables in determining the global spread of *B. anthracis*. Extreme cold and generally inhospitable circumstances delineate the boundaries of polar and desert regions, but temperate and tropical areas are constrained by more intricate criteria, including diurnal temperature range, annual temperature variations, and precipitation extremes. Comprehending these particular limiting criteria is essential for enhancing ecological niche models, forecasting future distribution changes due to climate change, and guiding focused surveillance and control measures for anthrax.

## Discussion

This study highlights the essential function of ecological niche modeling in comprehending the spatiotemporal dynamics of *B. anthracis* in the context of climate change scenarios. Our Maxent modeling clarifies the present and future habitat appropriateness for this bacterium, indicating substantial alterations in regional distribution that align with evolving climatic circumstances. These findings correspond with prior studies on other microorganisms that employed species distribution models (SDMs) to forecast habitat alterations resulting from climatic variability. Research on vector-borne diseases, including malaria and dengue fever, has illustrated that elevated temperatures and modified precipitation patterns can extend the geographic distribution of disease-carrying vectors, consequently heightening the risk of human exposure (Rocklöv and Dubrow, 2020; Franklinos et al., 2019). Our data suggest that classic anthrax-endemic areas may undergo changes in transmission dynamics, while new places could become conducive to *B. anthracis*, reflecting the adaptive responses seen in other diseases.

Moreover, the ecological niche of *B. anthracis* is shaped by numerous biotic and abiotic factors, as evidenced by our examination of bioclimatic variables. This aligns with prior studies that have recognized soil pH, temperature, and moisture as essential factors influencing pathogen survival and spread (Hugh-Jones and Blackburn, 2009; Carlson et al., 2019). The identification of high-risk locations for anthrax, especially in regions with evolving land-use patterns, highlights the need for integrated strategies that account for both climatic and human factors. Prior research has underscored analogous connections between alterations in land use and the introduction of diseases, suggesting that habitat fragmentation and intensified agriculture may heighten the risks of zoonotic spillover (Hansen et al., 2019; Walsh et al., 2022). Consequently, our findings augment the expanding corpus of literature that promotes proactive strategies in public health planning and surveillance to address these emerging hazards.

The predicted accuracy of our models, confirmed by stringent statistical approaches, aligns with the results of earlier studies employing machine learning techniques in epidemiology. Studies utilizing Maxent and other species distribution models (SDMs) have effectively predicted the distribution of several zoonotic diseases, including those causing Lyme disease and West Nile virus (Peterson et al., 2011; Merow et al., 2013). Our investigation reveals that the elevated AUC values demonstrate the model’s efficacy in differentiating appropriate habitats for *B. anthracis* spores, hence affirming the dependability of machine learning methodologies in forecasting pathogen distributions. Furthermore, the response curves generated by our model offer critical insights into the environmental thresholds affecting anthrax viability, similar to conclusions drawn in other microbial research that highlights the significance of species-environment interactions (Elith et al., 2011; Sofaer et al., 2019).

The ramifications of our findings are significant, especially considering the growing evidence connecting climate change to alterations in infectious disease patterns. The observed increases in habitat appropriateness for *B. anthracis* in certain regions reflect patterns identified in other research that have recorded analogous phenomena. The dissemination of *Borrelia burgdorferi*, the pathogen responsible for Lyme disease, has been strongly linked to climate-induced alterations in habitat suitability, resulting in heightened incidence rates in previously unaffected regions (Mora et al., 2022). Our research validates these findings, indicating that if climatic conditions enhance the survival of *B. anthracis*, the likelihood of anthrax outbreaks may increase, especially in areas that are presently ill-equipped to address such threats.

Furthermore, our work underscores the necessity of amalgamating ecological modeling with socio-economic variables to formulate thorough risk evaluations. The Coupled Model Intercomparison Project Phase 5 (CMIP) data we employed highlights the necessity for dynamic models that incorporate both climate and socio-economic trajectories, as demonstrated in other research evaluating the effects of climate change on infectious diseases (Eyring et al., 2016; Warszawski et al., 2021). Our research establishes a vital framework for targeted surveillance and intervention techniques by identifying prospective “gain” locations where habitat suitability for *B. anthracis* may augment. This proactive strategy is crucial for alleviating the dangers associated with zoonotic diseases, particularly in light of evolving land-use patterns that may exacerbate disease dynamics (Barro et al., 2016; Bezymennyi et al., 2021).

Our methodology and findings correspond with contemporary species distribution modeling research on fungal pathogens, including *Fusarium oxysporum* (Alkhalifah et al., 2023), *Aspergillus niger* (Alkhalifah et al., 2022), and *Phytophthora infestans* (Wang et al., 2023), all of which utilized MaxEnt modeling to forecast climate-induced distribution alterations. Consistent with our findings about the northward expansion potential of *B. anthracis*, research on *Pseudomonas syringae* (Khalaf et al., 2024) and other soil-borne bacterial diseases has revealed analogous range changes into higher latitudes in response to warmer scenarios. The alignment of our AUC values (0.831) with those documented in other microbial SDM investigations (often 0.80–0.90) substantiates our modeling methodology (Hosni et al., 2020; Chakraborty et al., 2022). Moreover, our recognition of temperature and precipitation thresholds as key limiting factors aligns with findings from vector-borne illness research, where analogous climatic variables influence pathogen viability and transmission dynamics (Kraemer et al., 2020; Carlson et al., 2022). The similarities among many pathogen types indicate that our climate-driven methodology encompasses essential ecological principles that regulate microbial dispersal amid global climatic change, hence enhancing the reliability of our forecast framework for *B. anthracis*.

The regions we identified as future climate suitability hotspots—including the Great Plains of North America, the Pampas of South America, and extensive pastoral areas of sub-Saharan Africa—are also major global livestock production centers where cattle density, grazing practices, and carcass management significantly modulate *B. anthracis* transmission risk (Barro et al., 2016; Kracalik et al., 2017). Intensive livestock production systems can create localized hotspots of anthrax risk through soil contamination from improperly disposed carcasses, concentrated animal feeding operations that facilitate rapid disease spread, and disrupted natural grazing patterns that alter soil-spore dynamics (Bezymennyi et al., 2021; Fasanella et al., 2013). Additionally, vaccination policies, surveillance capacity, and veterinary infrastructure vary dramatically across regions, creating disparities in actual disease risk that our climate-only models cannot capture (Hugh-Jones and Blackburn, 2009; Blackburn et al., 2017). Future integrated modeling efforts should incorporate livestock density data, production system classifications, and socioeconomic variables affecting disease control capacity to provide more actionable risk assessments. While our study establishes the foundational understanding of climatic suitability for *B. anthracis*, the translation of environmental suitability into actual outbreak risk requires explicit consideration of the complex livestock-soil-human interfaces that define modern anthrax epidemiology (Hansen et al., 2019; Walsh et al., 2022).

A notable downside of our work is the inherent limits of utilizing GBIF occurrence data for pathogen risk assessment, which may substantially influence the interpretation of our results. GBIF records for *B. anthracis* predominantly rely on molecular detection techniques, such as 16S rRNA gene sequencing, which verify the existence of bacterial genetic material but do not yield information about spore viability, concentration, or pathogenic potential (Turnbull, 2008; Carlson et al., 2019). Importantly, these records are unable to differentiate between viable, infectious spores that can induce disease and non-viable genetic material from dead spores or avirulent environmental isolates that lack the critical virulence plasmids pXO1 and pXO2 (Mock and Fouet, 2001; Koehler, 2009). This distinction holds epidemiological significance as the environmental persistence of *B. anthracis* DNA does not inherently correlate with the risk of active infection, which may result in an overestimation of disease threat in regions where genetic remnants exist without viable pathogen populations. Moreover, GBIF data are deficient in temporal information concerning spore persistence time and the environmental context of detection conditions, hence constraining our capacity to evaluate the sustainability of pathogen populations in forecasted suitable environments (Hugh-Jones and Blackburn, 2009).

Despite these data limitations, our study provides essential foundational knowledge for understanding the climate-driven environmental suitability patterns of *B. anthracis* on a global scale. The identification of climate-suitable regions remains crucial for guiding proactive surveillance strategies and resource allocation in the context of changing environmental conditions. These limitations underscore the importance of our modeling approach as a first step toward comprehensive risk assessment frameworks that can be refined through integration with more specific biological and epidemiological data on regional scale.

## Conclusion

In summary, our research enhances the comprehension of *B. anthracis* spread in a shifting environment, consistent with worldwide patterns in infectious disease ecology. Utilizing sophisticated machine learning methodologies and high-resolution climatic data, we offer critical insights that can guide public health policies and environmental management measures to mitigate the effects of anthrax and analogous zoonotic hazards in a warming climate.

## Data Availability

The original contributions presented in this study are included in this article/Supplementary material, further inquiries can be directed to the corresponding authors.
