# Supplementary material for: Spatiotemporal dynamics of Bacillus anthracis under climate change: a machine learning approach
Source: Front Microbiol. 2025 Oct 14;16:1659876. doi: 10.3389/fmicb.2025.1659876 (PMC12558879; doi:10.3389/fmicb.2025.1659876)
Supplement: Supplementary file 1 [file Supplementary_file_1.pdf]

Sp.	Latitude	Longitude
bacillus anthracis		
bacillus ant	7.786435	80.16971
bacillus ant	7.154037	80.18544
bacillus ant	7.081164	79.87931
bacillus ant	7.557552	80.67575
bacillus ant	6.90649	80.92137
bacillus ant	6.691737	80.30264
bacillus ant	6.532565	80.39906
bacillus ant	6.740258	81.01974
bacillus ant	7.084415	80.03094
bacillus ant	6.946866	80.78097
bacillus ant	7.196332	80.6729
bacillus ant	7.480134	80.61903
bacillus ant	6.141744	81.11938
bacillus ant	9.612738	80.33028
bacillus ant	6.063214	80.81098
bacillus ant	7.323314	80.77445
bacillus ant	6.808041	79.91335
bacillus ant	50.20432	80.47995
bacillus ant	-24.67	28.79
bacillus ant	-3	35.17
bacillus ant	-2.8	35.25
bacillus ant	-2.75	35.31
bacillus ant	43.9486	-79.4352
bacillus ant	-29	-50
bacillus ant	35.61139	126.4611
bacillus ant	34.8425	128.4431
bacillus ant	13.25	80.31
bacillus ant	47.42	7.08
bacillus ant	47.1132	8.5418
bacillus ant	50.8357	12.92922
bacillus ant	47.86789	17.26994
bacillus ant	-8.19	110.98
bacillus ant	-40	-72
bacillus ant	16.1856	-61.7447
bacillus ant	0.8197	11.4536
bacillus ant	-25.0619	46.8686
bacillus ant	-7.3056	147.1175
bacillus ant	-23.435	-45.2167
bacillus ant	-25.1008	-47.9275
bacillus ant	8.4503	4.6611
bacillus ant	-12.7014	130.9981
bacillus ant	33.9294	73.4047

bacillus ant	4.5533	-52.2092
bacillus ant	-0.0586	114.005
bacillus ant	42.0311	128.4314
bacillus ant	-24.0653	-48.9878
bacillus ant	31.9453	5.3953
bacillus ant	11.12	75.52
bacillus ant	11.12	75.49
bacillus ant	23.72	92.71
bacillus ant	7.16	3.33
bacillus ant	-25.94	29.2
bacillus ant	7.19	3.45
bacillus ant	7.39	3.87
bacillus ant	37.13	-6.57
bacillus ant	23.28	87.08
bacillus ant	31.629	74.2833
bacillus ant	21	85
bacillus ant	24.73	93.97
bacillus ant	26.75	94.22
bacillus ant	19.3	84.24
bacillus ant	-29.82	25.35
bacillus ant	38.07	-1.07
bacillus ant	1.2	103.77
bacillus ant	41.7151	44.8271
bacillus ant	51.14	51.22
bacillus ant	-19.0372	15.5537
bacillus ant	-19.0313	15.5487
bacillus ant	51.2016	11.8357
bacillus ant	52.9905	11.6795
bacillus ant	49.81	50.67
bacillus ant	6.29	8.11
bacillus ant	30.15	71.52
bacillus ant	35.57	138.48
bacillus ant	6.61	3.39
bacillus ant	8.17	77.43
bacillus ant	6.47	3.38
bacillus ant	31.37	74.55
bacillus ant	16.3	80.44
bacillus ant	24.2	84.87
bacillus ant	25.5	82.9
bacillus ant	43.46	87.36
bacillus ant	-26.69	27.03
bacillus ant	12.82	79.69
bacillus ant	47.9539	11.9845
bacillus ant	26.61	91.58

bacillus ant	-30.4	-35
bacillus ant	11.23	75.79
bacillus ant	51.54	46
bacillus ant	-28.55	26.1
bacillus ant	-26.29	26.06
bacillus ant	28.08	77.12
bacillus ant	62.03	29.98
bacillus ant	71.2	-156.41
bacillus ant	35.9336	-88.7224
bacillus ant	1.627778	-75.9047
bacillus ant	1.607944	-75.8868
bacillus ant	1.701889	-75.8993
bacillus ant	1.604944	-75.8808
bacillus ant	1.670667	-75.9037
bacillus ant	1.717889	-75.9033
bacillus ant	1.624278	-75.9012
bacillus ant	1.679222	-75.9034
bacillus ant	1.697194	-75.905
